# Comparison of the Efficacy of Amitriptyline and Topiramate in Prophylaxis of Cyclic Vomiting Syndrome

**Published:** 2019

**Authors:** Zahra BAGHERIAN, Omid YAGHINI, Hossein SANEIAN, Shervin BADIHIAN

**Affiliations:** 1 Child Growth and Development Research Center, Isfahan University of Medical Sciences, Isfahan, Iran

**Keywords:** Amitriptyline, Topiramate, Cyclic vomiting syndrome, RCT, Prophylaxis

## Abstract

**Objectives:**

Cyclic vomiting syndrome (CVS) is a chronic functional gastrointestinal disorder with no certain treatment. We aimed to compare the efficacy of amitriptyline and topiramate on prophylactic therapy of CVS.

**Materials & Methods:**

This randomized clinical trial (registration number: IRCT2015102316844N2) was conducted during 2016 in Isfahan, central Iran. The inclusion criteria were CVS patients (based on Rome III) aging 3-15 yr with normal physical examination, no metabolic disorder, and no gastrointestinal obstruction or renal impairment. Recruited patients were divided into two groups of amitriptyline (1 mg/kg/d) and topiramate (1-2 mg/kg/d) and were followed for 3-months. The outcome was evaluated by comparing severity of attacks (monthly frequency and duration of attacks) before and after intervention.

**Results:**

Thirty-six children entered each group and two patients left the amitriptyline group. Patients and disease characteristics were similar between groups before intervention (*P*>0.05). The frequency of attacks (standard deviation) after intervention in amitriptyline and topiramate group was 0.91 (0.40) and 1.07 (0.55), respectively (*P*=0.368) and the duration of attacks (SD) after intervention were 3.43 (2.46) and 4.90 (3.03), respectively (*P*=0.017). Twenty-three patients (68%) in amitriptyline group and 14 patients (39%) in topiramate group stopped having attacks after intervention (*P*=0.016).

**Conclusion:**

Amitriptyline is a better choice to reduce severity of CVS attacks compared to topiramate, in a short-term evaluation. Studies with longer follow-up are required to investigate these findings in a longer period.

**What is known?**

- Cyclic vomiting syndrome is a chronic disease characterized by recurrent self-limited nausea and vomiting episodes with symptom-free intervals.
- There is no certain treatment for cyclic vomiting syndrome yet.
- Amitriptyline, imipramine, topiramate, propranolol, erythromycin, cyproheptadine, and combination of L-Carnitine and CoQ10 have been suggested to be effective in treatment of cyclic vomiting syndrome.

**What is new?**

- Amitriptyline can be used as an effective treatment in children with cyclic vomiting syndrome with response rate of >90% in our series.
- Amitriptyline has a superiority on topiramate in prophylactic treatment of cyclic vomiting syndrome.
- Topiramate is not an effective drug for treatment of cyclic vomiting syndrome.

## Introduction

Cyclic vomiting syndrome (CVS) is a chronic idiopathic disease characterized by recurrent self-limited nausea and non-bilious vomiting episodes lasting from few hours to few days with symptom-free intervals between them (1, 2). CVS is diagnosed based on Rome III criteria: stereotypical episodes of vomiting regarding onset (acute) and duration; three or more discrete episodes in the prior year; absence of nausea sensation and vomiting in intervals and absence of metabolic, gastrointestinal and central nervous system disorders (3).

CVS mostly begins in childhood and is one of the most important causes of reversible periodic nausea in children (4). It is more prevalent among females and its occurrence increases in physical and mental stresses (5-7). CVS attacks are characterized by severe non-bilious vomiting (up to 6 times/h), and with debilitating nausea that can cause severe dehydration and may lead to intravenous therapy (1). Other symptoms include pallor, lethargy, nausea, abdominal pain, loss of appetite, photophobia and headache (1). CVS is usually misdiagnosed with other disorders since there are no specific symptoms and diagnostic paraclinical tests for the condition (8).

The underlying mechanisms causing this disorder are not well understood, however, some theories are suggested in the literature: dysfunctional brain-gut interaction; corticotrophin releasing factor disorder; abnormal function of the autonomic nervous system; mitochondrial dysfunction due to DNA mutations that cause a deficiency of cellular energy production; and heightened hypothalamic stress response leading to nausea (1, 2, 5, 8). Moreover, the relationship between CVS and migraine is suggested from a long time ago considering their similarities in clinical features and reasonable successes in treatment of CVS with anti-migraine medication (9, 10).

CVS attacks may cause adverse effects including esophagitis, hematemesis, intracellular electrolyte decrease, hypertension, and syndrome of inappropriate antidiuretic hormone secretion (4). This disorder can cause children to be absent at school, parents to be absent from work, leading to socio-economic losses, and incurring medical expenses for families (4, 7). In addition, children with CVS experience 5%-15% decrease in their quality of life (6, 7). These all indicate the importance of finding an effective treatment for CVS.

Various drugs have been experimented for this disorder. In children, amitriptyline, imipramine, topiramate, propranolol, erythromycin, cyproheptadine, and combination of L-Carnitine and CoQ10 have been suggested (2, 5, 6). Amitriptyline is one of the most common drugs with a favorable response (11). Moreover, topiramate is an anti-migraine medication (2, 12, 13) with responder rates as high as 94% (12). Despite these findings, there is not a reliable treatment protocol for CVS yet and more clinical trials need to be performed on this issue (5). 

In this study, we aimed to compare the efficacy of topiramate and amitriptyline on CVS prophylaxis.

## Materials & Methods

This randomized clinical trial was conducted in Imam Mousa Sadr Clinic, Isfahan University of Medical Sciences, Isfahan, Iran and a private Gastroenterology Clinic from Feb 2016 to Aug 2016. Children 3-15 yr old referred to the mentioned clinics with possible diagnosis of CVS were initially considered for participation and those with the following criteria were enrolled: 1) Having the diagnostic criteria of cyclic vomiting syndrome based on Rome III (3); 2) Normal neurological and developmental physical examination, 3) Absence of any metabolic disorder, 4) Absence of any gastrointestinal obstruction or renal impairment. 

Patients who refused to fill informed consent to participate in the study and those who decided to leave the study for reasons other than adverse drug reactions were excluded. Informed consent was obtained from patients or their parents prior to the study. The study was approved by regional Bioethics Committee of Isfahan University of Medical Sciences and was registered in Iranian Registry of Clinical Trials (registration number: IRCT2015102316844N2).

The diagnosis was confirmed by a Pediatric Neurologist or a Pediatric Gastroenterologist (coauthors of the study). A complete medical history was taken from patients and they underwent a thorough physical examination (including neurological examination). Besides, data on frequency and severity of their CVS attacks, history of hospitalization, and possible complications were collected retrospectively. 

After enrollment of patients, they were divided into two groups randomly using block randomization and each block was allocated to one of the groups of amitriptyline or topiramate using numbered envelopes thereafter. One group was treated with 1 mg/kg/d of amitriptyline (produced by Pars Daru company in Iran) and the other group was treated with 1-2 mg/kg of topiramate (produced by Pars Daru company in Iran) twice a day.

Groups were followed for 3 months after starting the medication, looking for any response to the medication. They were visited regularly during this period every two weeks. The frequency and duration of attacks and adverse drug reactions were asked in each visit as the primary outcome of the study. The mean frequency and duration of attacks in the three months of intervention were compared then to the frequency and duration of attacks before intervention. We assumed patients who stopped having attacks at least in the last month of follow-up as vomit-free patients and compared them between study groups. Moreover, patients who had ≥50% reduction in frequency or duration of attacks were compared between two groups. During the study, patients were recommended to refer to clinics if they experienced any adverse drug reactions.

Descriptive statistics were used to describe data as frequencies for categorical variables and mean (standard deviation) for interval variables. To compare means, independent sample *t*-test, Mann-Whitney test, and Wilcoxon signed-rank test were used when applicable. Chi-square test was also used to compare categorical data. Statistical analysis was performed using SPSS 19 (Chicago, IL, USA) and a* P*-value of less than 0.05 was considered significant.

## Results

Overall, 72 children were enrolled in the study initially and 70 of them remained as the study population till the end, ranging from 4 to 13 yr old. Patients were divided into two groups of 36 participants initially, and 2 subjects from amitriptyline group decided to leave the study to continue treatment in another medical center. The mean age (yr) (standard deviation) of participants in the amitriptyline and topiramate group was 8.30 (2.12) and 8.10 (2.36), respectively (*P*=0.705). In the amitriptyline group 15 (44%) subjects were male and in the topiramate group, 17 (47%) were male (*P*=0.794). 

Table 1 shows the disease characteristics in each study group before and after intervention. With respect to the efficacy of each drug, in amitriptyline group the mean monthly frequency of attacks and the duration of attacks both decreased significantly after intervention (both *P*-values<0.001). Same results were observed in topiramate group regarding the decline in the mean monthly frequency and duration of attacks after intervention (both *P*-values<0.001).

**Table 1:** Comparison of attack characteristics and overall outcome before and after intervention between two groups of topiramate and amitriptyline

***P*** -value*		**Amitriptyline**		**Topiramate**		**Category**
		**Median**	**Mean ± SD** ^1^	**Median**	**Mean ± SD**	
Intervention	Frequency of attacks^2^	1	1.50 ± 0.70	1	1.56± 0.66	0.603
	Duration of attacks^3^	6	6.93 ± 3.30	5.5	6.26 ± 3.41	0.296
After Intervention	Frequency of attacks	1	1.07 ± 0.55	1	0.91 ± 0.40	0.368
	Duration of attacks	4	4.90 ± 3.03	3	3.43 ± 2.46	0.017
	Number of vomits per hour*	2	3.7 ± 3.44	2.5	2.5 ± 1.58	0.309
	Number of vomits per attack**	6	6.53 ± 3.90	6	7.33 ± 3.68	0.581

1 Standard Deviation;

2 Number per month;

3 Hour per attack

* *P*-values are obtained from comparison of means

** Values of cases with complete remission are not reported

There was no statistically significant difference between frequency and duration of attacks in two groups before intervention. After intervention, the mean monthly frequency of attacks (SD) in amitriptyline and topiramate group was 0.91 (0.40) and 1.07 (0.55), respectively, and the difference was not statistically significant (*P*=0.368); however, the mean duration of attacks (SD) after drug administration was lower in amitriptyline group (3.43 (2.46) compared to 4.90 (3.03), *P*=0.017). We also compared the mean number of vomits per attack and the mean number of vomits per hour between two study groups after intervention and found no statistically significant difference (*P*=0.309 and *P*=0.581, respectively).

Twenty-three patients (68%) in amitriptyline group and 14 patients (39%) in topiramate group were vomit-free after treatment (*P*=0.016). Moreover, 27 patients (79.4%) in amitriptyline group and 16 patients (44.4%) in topiramate group had ≥50% improvement after intervention (*P*=0.003). Two patients in amitriptyline group experienced constipation during the study and 

none of the patients in topiramate group reported any adverse drug reactions (Table 2).

**Table 2:** Comparison of remission rates and side effects between two study groups

**Category**		**Topiramate (No (%))**	**Amitriptyline (No (%))**	***P*** **-value**
Complete remission	Yes	14 (38.9%)	23 (67.6%)	0.016
	No	22 (61.1%)	11 (32.4%)	
≥50% remission	Yes	16 (44.4%)	27 (79.4%)	0.003
	No	20 (55.6%)	7 (20.6%)	
Side effects	Yes	0	2 (5.9%)*	0.140

* Two cases with constipation

## Discussion

Amitriptyline is a tricyclic antidepressant (TCA) reported in previous studies (6, 14, 15) as one of the most effective treatments for CVS patients. The efficacy of this drug has been reported 52% to 93% (6, 14, 16, 17, 18). In contrast to amitriptyline, topiramate is studied in fewer studies before and only small number of reports are available suggesting topiramate as an appropriate and effective choices for prophylactic treatment of CVS (6, 12, 13).

In the present study, patients showed a favorable response to both amitriptyline and topiramate regarding decrease in frequency and duration of attacks. We observed 39% full remission after topiramate administration and 68% full remission after amitriptyline administration. Andersen et al reported 73% of patients with complete remission and 18% with partial remission after a follow-up of 5 months to 10 yr (19). Moreover, in a study, 93% of their patients experienced decreased symptoms and 26% stopped having attacks after 3 months of treatment with amitriptyline (17). In a randomized clinical trial conducted by our group, we found full remission among 65.6% of patients receiving amitriptyline after 6 months of follow-up (18). Additionally, a prospective Iranian study on children with CVS reported effectiveness of amitriptyline in 56% of their patients (20). Our findings on efficacy of amitriptyline seem to be consistent with these reports although they had different settings, methods, and follow-up durations.

On the other hand, there are very limited data available on the efficacy of topiramate on CVS prophylaxis. Boles et al. administered topiramate for two patients with refractory attacks while being treated with other drugs and they both had resolution of attacks (2). Moreover, another study showed about 15% complete remission and 45% partial remission in 18 patients being treated with topiramate (13). A recent retrospective study evaluated 16 pediatric patients treated with topiramate for at least 12 months and reported freedom of attacks in 81% of them and >50% decrease of attacks in 13% of them (12). Although we had decreased frequency and duration of attacks in patients treated with topiramate, only 39% of our cases stopped having attacks. However, this is hard to compare our findings with previous ones considering the short follow up of our cases and retrospective design of previous studies.

There are no previous studies comparing the efficacy of topiramate and amitriptyline on prophylactic therapy of CVS, however, a recent meta-analysis on migraine medications showed that amitriptyline was weakly superior to other drugs including topiramate (21). Although the relationship between CVS and migraine has been suggested since long time ago (9), these findings may not be applicable for patients with diagnosis of CVS. In this study, we observed a more favorable response to amitriptyline compared to topiramate. The duration of attacks was decreased more in amitriptyline group and more patients stopped having attacks in amitriptyline group either. Therefore, we suggest the superiority of amitriptyline on topiramate in prophylactic therapy of CVS.

This study had some limitations: first, considering the design and methodology of the study we were able to enroll limited number of patients which may affect our results. Second, we did not evaluate various doses of drugs in the patients and a single dose was only administered. Third, we evaluated patients after three months of therapy which is a short follow up period compared to most previous studies and administered drugs may have different long-term therapeutic effects. Therefore, we believe a different outcome may be observed in similar studies with longer follow up periods. Fourth, we collected data considering frequency and duration of attacks before intervention based on patients’ medical history and parents’ declaration. Therefore, a recall bias may have affected our results and overestimated data regarding attack characteristics before intervention. Fifth, we visited patients regularly after intervention caused a placebo effect and thus a better overall outcome in both groups. Despite these limitations, this is the first clinical trial comparing the efficacy of topiramate and amitriptyline in children with CVS.

**In conclusion,** amitriptyline is a better choice to reduce severity of CVS attacks compared to topiramate, in a short-term evaluation. Studies with longer follow-up are required to investigate these findings in a longer period. There is still lack of evidence on this issue, especially clinical trials, and further studies are recommended to confirm our findings.

## Clinical trial registration

The study was registered in Iranian Registry of Clinical Trials (www.IRCT.ir) with registration number: IRCT2015102316844N2
